# Cytohistological correlation of early changes in spontaneously infarcted fibroadenoma—a rare case report

**DOI:** 10.3332/ecancer.2012.257

**Published:** 2012-06-12

**Authors:** HT Kamra, PA Gadgil, SA Chaware, AB Kolhe, PW Sakinlawar

**Affiliations:** Department of Pathology, Government Medical College, Latur-413512, Maharashtra, India

## Abstract

Fibroadenoma is the most commonly diagnosed benign tumour in adolescents and young women but spontaneous infarction in fibroadenoma is rarely seen. We report here a case of spontaneously infracted fibroadenoma in a 20 year old unmarried female without history of trauma or previous fine needle aspiration. It is important to recognize this entity because microscopic findings in an infarct are influenced by the duration of the infarct and on cytology it mimics mastitis, duct ectasia and even carcinoma.

## Introduction

Fibroadenomas are benign tumours arising from the epithelium and stroma of the terminal duct lobular unit and constitute 20% of all the benign breast lumps [1]. Risk factors include the use of oral contraceptives before the age of 20 [2] and Epstein–Barr virus infections in immunosuppressed patients [3]. Secondary changes include hyalinization, calcification, ossification, prominent myxoid changes, apocrine or squamous metaplasia, sclerosing adenosis, and lactational changes [4], though spontaneous infarction is a very rare complication. In Haagensens’ review of fibroadenoma, the incidence of spontaneous infarction was 5 out of 1000 cases (0.5%), and 3 of these 5 patients were pregnant or lactating for the first time [5]. Here we report a case of spontaneously infarcted fibroadenoma in a 20-year-old female. The cytohistology is in agreement with the early changes of a haemorrhagic infarct of a fibroadenoma.

## Case report

**Figure 1:**
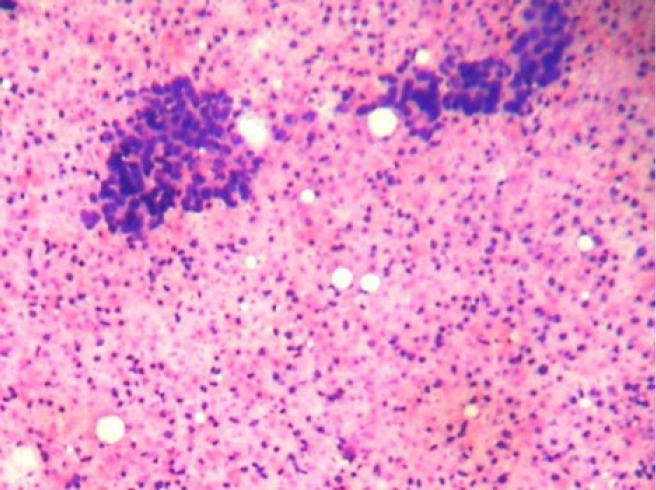
Cellular smears showing tight clusters of duct epithelial cells and myoepithelial cells with abundant ghost epithelial cells (4000 × 3000 pixels)(H & E stain).

**Figure 2:**
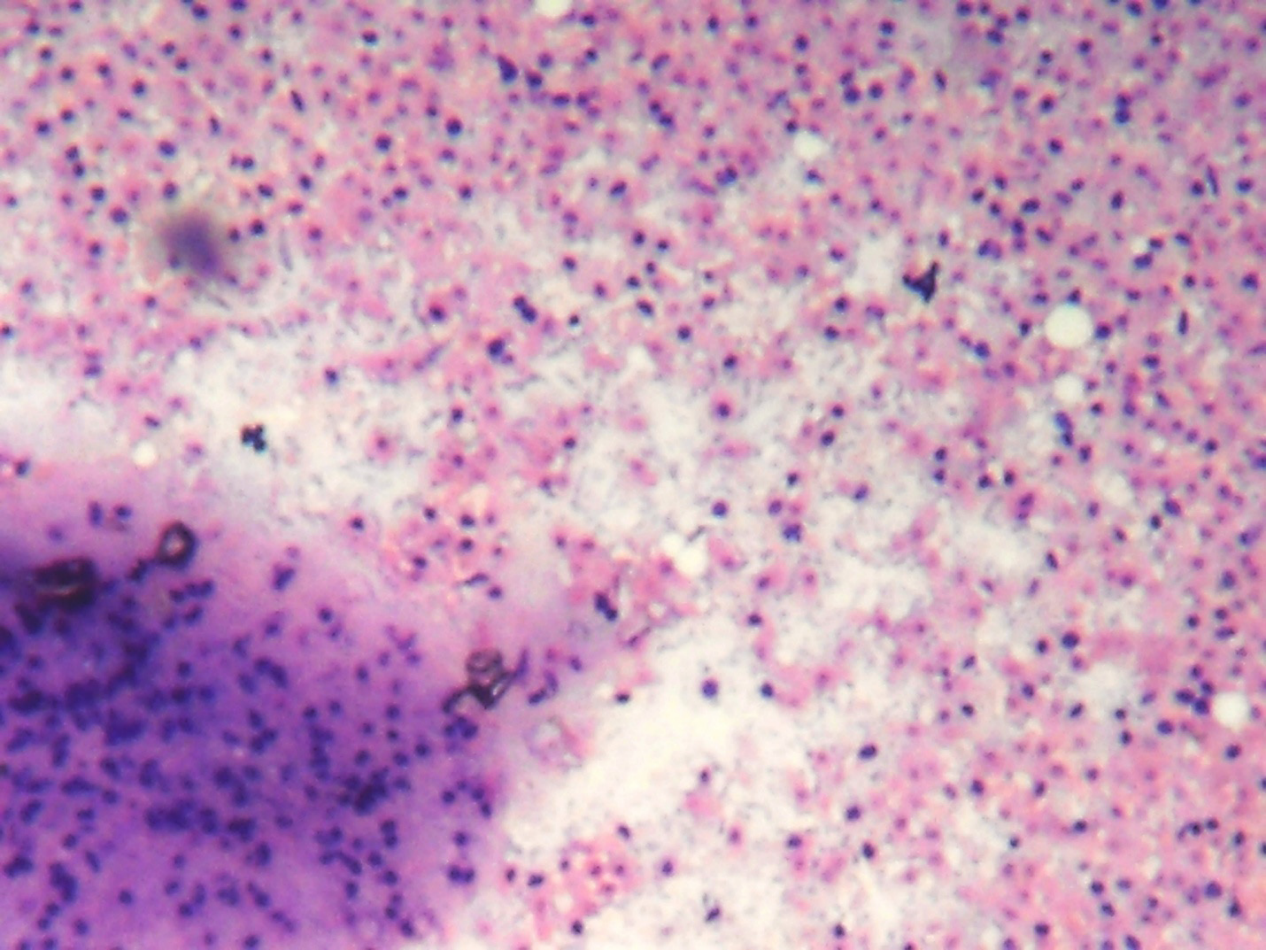
(FNAC SMEAR) Cellular smears showing fibromyxoid stroma in the background of abundant ghost epithelial cells(4000 × 3000 pixels)(H & E stain).

**Figure 3:**
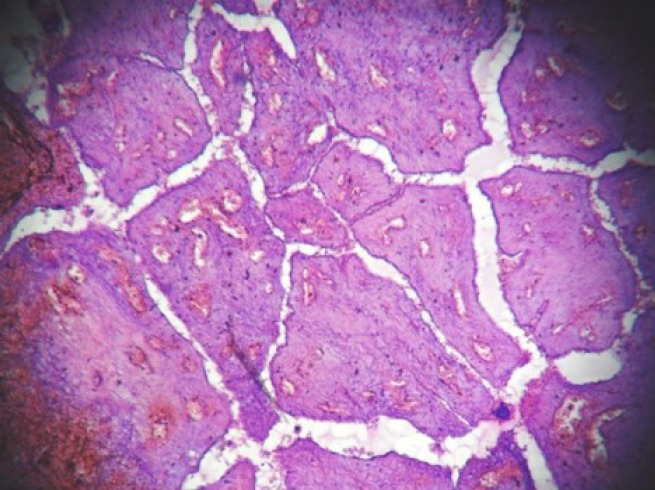
Hemorrhagic infarct in intracanalicular type of fibroadenoma (4000 × 3000 pixels)(H & E stain).

**Figure 4:**
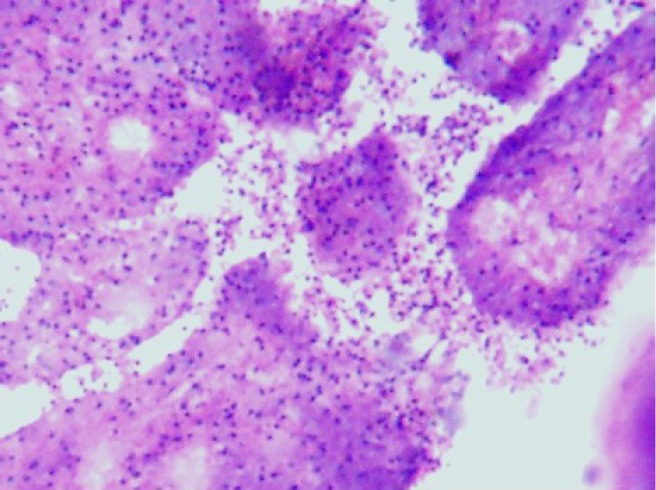
Desquamation of lining epithelial cells which appeared as the ghost cells in the cytology smears (4000 x 3000 pixels)(H & E stain).

**Figure 5:**
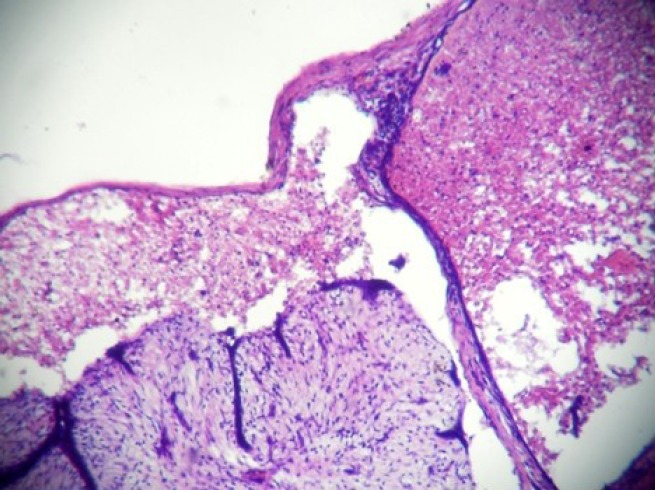
Complete disintegration of architecture forming cystic space containing singly scattered stromal and ductal cells (ghost epithelial cells) (4000 × 3000 pixels)(H & E stain).

A 20-year-old unmarried female presented a well-circumscribed mobile lump in the upper outer quadrant of her left breast. The lump had gradually increased in size over the last six months and had started to be painful in the last few days. On clinical examination, the lump measured 5.5 cm × 4 cm, and the skin over the lump was not attached to the mass and showed no signs of inflammation. The patient presented this for the first time to the surgery outpatient department. Moreover, there was no history of trauma and no previous fine needle aspiration cytology (FNAC) had been performed. The lump was diagnosed clinically as a fibroadenoma, and the FNAC was carried out with a 5-ml syringe using a 24-gauge needle. Smears were dried, fixed, and stained with hematoxylin and eosin and then evaluated by the pathologist. The smears were highly cellular; most of the cells were scattered individually and had hyperchromatic round/oval nuclei with smudged chromatin and eosinophilic cytoplasm. There were only a few tight clusters of ductal epithelial cells with myoepithelial cells, and a few focal areas of fibromyxoid stroma (Figures 1 and 2). There was no evidence of any necrosis, and no inflammatory cells or red blood cells were present. A diagnosis of fibroadenoma with secondary squamous metaplasia was reported after the FNAC, and an excision biopsy was carried out. The removed tissue was fixed in 10% buffered formalin overnight. On macroscopic inspection, the specimen was found to be an encapsulated grey white mass measuring 5.2 cm × 4 cm × 3.5 cm. A grey brown area measuring 4 cm × 4 cm × 3.5 cm with small cystic areas was evident in the cross-sectional view of the specimen. The remaining part of the specimen was firm, grey white with a single small cystic area. Representative multiple sections were prepared from the grey brown areas, the cystic areas, and the firm grey white area. Tissue samples, measuring 4–5 mm in size, were fixed in the 10% neutral buffered formalin, processed under standardized conditions for paraffin embedding. Sections of 3–4 µm were cut and stained with hematoxylin and eosin using the standard procedures. On microscopic examination, sections from the grey white area indicated an intracanalicular type of fibroadenoma with cleft-like spaces. In addition, sections from grey brown areas revealed areas of ischemic hemorrhagic necrosis within the fibroadenoma. The outlines of the intracanalicular pattern were still retained along with congested small vessels and areas of haemorrhage (Figure 3). The epithelium lining the cleft-like spaces was desquamated and lying as individual cells in the cleft spaces (Figure 4). Few lobules showed complete disintegration of ductal and stromal cells. The ductal cells were round to polygonal in shape, and stromal cells were spindle shaped. Both these cell types were lying singly in a cystic space lined by the ductal epithelial cells (Figure 5). No inflammatory cells were evident. The histological diagnosis was fibroadenoma with subtotal infarction.

## Discussion

Fibroadenoma is the most commonly diagnosed benign tumour on fine aspiration cytology. The age distribution ranges from childhood to 70 years, but is more common in adolescents and young women [6]. Delarue and Redon (1949) were the first to describe the infarction of fibroadenoma [7]. Since spontaneous infarcts are frequently associated with pregnancy and lactation, they are commonly reported in women younger than 35 years [8], but a rare case of a spontaneous infarction in a 58-year-old postmenopausal women has been reported [9]. Infarction of fibroadenoma has been reported after FNAC [10] and sporadic cases of infarction have occurred in patients on anticoagulant drugs [11], but the patient reported here had no such history. Cytological analysis must differentiate infarcted fibroadenoma from mastitis and duct ectasia including carcinoma [12]. In the tissue smears reported here, no necrotic or inflammatory regions were evident. The diagnosis of carcinoma should be considered only when the viable tumour tissue is identified along with necrosis [13]. Some authors described organized or organizing thrombi in areas of infarction or in adjacent tissue [14], but no evidence of thrombo-occlusive or inflammatory vascular diseases was observed in this patient.

Microscopic findings in an infarct are influenced by the duration of the infarct. Haemorrhage and ischemic degeneration with little or no inflammation as seen in our case characterize early lesions. It is in the later stages of infarction that a loss of nuclear detail, pallor, zones of granulation tissue with variable degrees of inflammation, hemosiderin deposition, and fibrosis are seen [8]. We were unable to recognize ghost epithelial cells in cytological smears due to the absence of necrosis and inflammation, although after histopathological staining these cells could be clearly identified. We therefore conclude that the case reported here represents a fibroadenoma with early changes resulting from a haemorrhagic infarct without any etiologic factor. Although an uncommon complication, spontaneous infarction should always be considered in differential diagnosis in fine needle aspiration of breast lumps.
